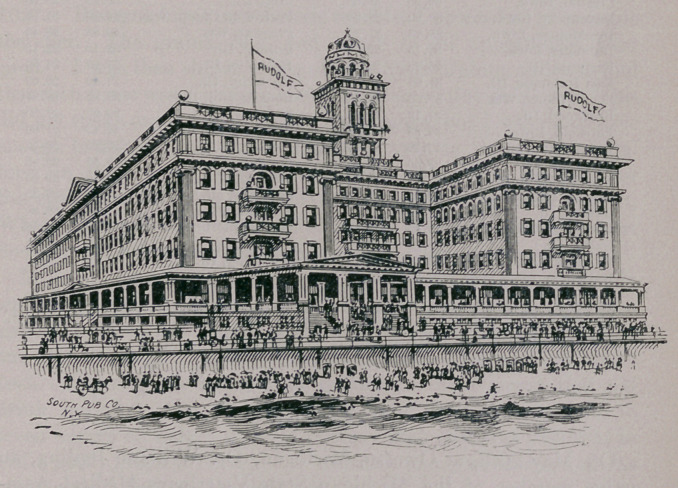# A. V. M. A. Annual Meeting

**Published:** 1901-07

**Authors:** 


### A. V. M. A. ANNUAL MEETING.

THE Committee of Arrangements for the annual meeting of the American Veterinary Medical Association report that they have made most satisfactory plans for the headquarters of the Association, and the place of meeting for the convention at THE NEW RUDOLF. The hotel has given rates from $2.50 per day, and for two people in a room at $5, including a bath room. The beach for sea-bathing is directly in front of the hotel. The accompanying illustration is an excellent view of the hotel.

The committee also report that the railroad rates promise to be most favorable, especially from the West. These arrangements are not completed, but as a guide to what they expect to obtain they report that we will be given the benefits offered by the Chicago, Burlington, and Quincy Railroad Company to the Pan-

American Exposition, Buffalo, N. Y., June 1, to November 1, 1901, as follows:

For the above occasion tickets may be sold at the rates and on the conditions shown below :

### Thirty Days Tickets.

1. Rates via Chicago and L. S. and M. S. Ry., or M. C. R.R.
2. Rates via all other direct routes.

| | 1 | 2 | | 1 | 2 |
|---|---|---|---|---|---|
| Abington, Ill. | $28.00 | $28.70 | Keithsburg, Ill. | 30.20 | 30.20 |
| Afton Junction, Iowa, | 38.90 | 38.90 | Knoxville, Iowa, | 36.45 | 35.95 |
| Albia, Iowa, | 35.25 | 35.25 | La Crosse, Wis. | 36.00 | 33.60 |
| Amboy, Ill. | 27.70 | 25.55 | La Salle, Ill. | 25.80 | 25.50 |
| Aurora, Ill. | 25.65 | 23.25 | Libertyville, Iowa, | 33.25 | 33.25 |
| Beardstown, Ill. | 29.20 | 29.20 | Malvern, Iowa, | 42.95 | 41.00 |
| Burlington, Iowa, | 30.40 | 30.40 | Mendota, Ill. | 26.75 | 25.00 |
| Bushnell, Ill. | 28.70 | 28.70 | Monmouth, Ill. | 29.00 | 29.00 |
| Canton, Ill. | 27.20 | 27.20 | Omaha, Neb. | 43.90 | 41.50 |
| Chicago, Ill.[1] | 23.40 | 21.00 | Oregon, Ill. | 27.75 | 25.35 |
| Clinton, Iowa, | 30.25 | 27.85 | Orion, Ill. | 30.20 | 28.95 |
| Chapin, Ill. | 29.20 | 29.20 | Ottawa, Ill. | 25.80 | 24.80 |
| Chariton, Iowa, | 36.70 | 36.70 | Ottumwa, Iowa, | 34.00 | 34.00 |
| Council Bluffs, Iowa, | 43.40 | 41.00 | Peoria, Ill.[1] | 25.80 | 25.80 |
| Creston, Iowa, | 39.55 | 39.55 | Polo, Ill. | 28.35 | 25.95 |
| Davenport, Iowa, | 30.20 | 28.95 | Prairie du Chien, Wis. | 34.30 | 31.90 |
| Des Moines, Iowa, | 38.15 | 37.25 | Prescott, Wis. | 40.85 | 38.45 |
| Dubuque, Iowa, | 32.15 | 29.75 | Quincy, Ill. | 32.15 | 32.15 |
| Earlville, Ill. | 26.75 | 24.50 | Rochelle, Ill. | 27.00 | 24.60 |
| East Dubuque, Ill. | 31.65 | 29.25 | Rockford, Ill. | 27.40 | 25.00 |
| East Winona, Wis. | 37.05 | 34.65 | Rock Island, Ill. | 30.20 | 28.95 |
| Elmwood, Ill. | 27.10 | 27.10 | Savanna, Ill. | 29.90 | 27.50 |
| Fairfield, Iowa, | 32.85 | 32.85 | Shenandoah, Iowa, | 42.45 | 41.00 |
| Farmington, Ill. | 27.20 | 27.20 | Sterling, Ill. | 28.65 | 26.25 |
| Forreston, Ill. | 28.50 | 26.10 | Streator, Ill. | 25.80 | 25.15 |
| Fort Madison, Iowa, | 31.30 | 31.30 | St. Joseph, Mo. | 39.75 | 39.75 |
| Fulton, Ill. | 29.95 | 27.55 | St. Louis, Mo.[1] | 31.30 | 29.25 |
| Galena, Ill. | 31.15 | 28.75 | St. Paul, Minn. | 41.80 | 38.80 |
| Galesburg, Ill. | 28.35 | 28.35 | Winona, Minn. | 37.20 | 34.80 |
| Galva, Ill. | 28.35 | 27.70 | Wyanet, Ill. | 27.10 | 26.35 |
| Hannibal, Mo. | 32.15 | 32.15 | Wyoming, Ill. | 27.10 | 27.10 |
| Indianola, Iowa, | 38.30 | 37.90 | | | |

It is expected that a return rate from Buffalo to Atlantic City can be obtained for $10.

## Figures and Tables

**Figure f1:**